# Toolpath Planning and Generation for Multi-Stage Incremental Forming Based on Stretching Angle

**DOI:** 10.3390/ma14174818

**Published:** 2021-08-25

**Authors:** Hu Zhu, Guixi Cheng, Dongwon Jung

**Affiliations:** 1 College of Mechanical and Electrical Engineering, Shenyang Aerospace University, Shenyang 110023, China; cgx1213@126.com; 2 Department of Mechanical Engineering, Jeju National University, Jeju-si 63243, Korea

**Keywords:** incremental forming, single point incremental forming, multi-stage forming, forming, toolpath

## Abstract

To solve the problems that exist in the multi-stage forming of the straight wall parts, such as the sheet fracture, uneven thickness distribution, and the stepped feature sinking, a new forming toolpath planning and generation method for the multi-stage incremental forming was proposed based on the stretching angle. In this method, the parallel planes that were used for forming toolpath generation were constructed by using the stretching angle so that the distances between the parallel planes and the forming angles were gradually reduced. This makes the sheet material flow become changed and the thickness thinning is relieved. The software system for the toolpath generation was developed by using C++, VC++, and OpenGL library. In order to verify the feasibility of the proposed method, numerical simulation and forming experiments were carried out for the single stage forming, the traditional multi-stage forming, and multi-stage forming based on the proposed forming toolpath, using 1060 aluminum sheets. The comparative analysis of the thickness distribution, profile curve, strain curve, and sheet material flow shows that the proposed method is feasible, and the profile dimension accuracy is better, the thickness distribution is more uniform, and the sinking and bulging are significantly reduced. The formed sheet part with the stretching angle of 15° has higher dimensional accuracy, smaller bottom subsidence, and larger thickness than that of the stretching angle 5°.

## 1. Introduction

Sheet metal forming technology is continuously developing, with wide application and the improvement of plasticity of the sheet metal [1]. As a new sheet metal forming method, the sheet metal Computer Numerical Control (CNC) incremental forming has better formability than other conventional forming technology [2]. The forming tool gradually extrudes the sheet metal according to the preprogrammed toolpath to make it deformed so as to obtain a three-dimensional geometric model [3]. Because of the flexibility and dieless characteristics, this technology has attracted much attention and has been applied in various fields, such as medical treatment, aerospace, automobile manufacturing, etc., and also plays an extremely important role in more-variety-and-less-batch production [4,5].

In industrial production, the demand for asymmetric parts and straight wall parts is increasing [6]. However, the single stage forming has some limitations in forming the straight wall parts with a large forming angle [7]. In order to solve the problem of the straight wall parts forming, many scholars proposed to add intermediate forming stages on the basis of the single stage forming to improve the formability of the single stage forming, that is, the multi-stage forming [8]. The research results show that the number of intermediate forming stages, forming toolpath, forming strategy, and forming direction of the multi-stage forming have influence on the thickness distribution and geometric accuracy of the straight wall parts [9].

Mercedes et al. [3] compared forming quality between the multi-stage forming and single stage forming. The authors highlighted that the multi-stage forming can improve the geometric accuracy, but the thickness of the sheet metal part with multi-stage forming is not uniform. Pranav and Jacob [10] have successfully formed the C-channel for the aircraft fuselage vibration test by adopting the multi-stage forming strategy. Pranav et al. [11] presented a straight wall part forming method based on six stage incremental forming, which can improve the thickness distribution and reduce geometrical errors in critical areas. Kurra et al. [12] studied the thickness distribution, strain, and geometric accuracy of the straight wall part with a forming angle of 85°. They highlighted that the thickness cannot be predicted with the increase in the number of intermediate forming stage, the strain paths are changed into biaxial tensile direction, and the phenomenon of sinking occurs in each forming stage. Wu [13] proposed a new parameterized multi-stage forming strategy to reduce the stepped features in the forming of the straight wall part and also highlighted that the mismatch between the tool radius and vertical displacement is the main factor of the thickness distribution and stepped features. Nirala [8] proposed an effective multi-stage forming strategy to eliminate the sinking phenomenon and discussed the number of intermediate forming stages required by avoiding the overlapping forming of the sheet metal. Dai et al. [14] studied the multi-stage forming of sheet metal parts with a non-axisymmetric complex cross section by using the compensated intermediate forming stage toolpath so as to improve the geometric accuracy. Zhao and Meehan [15] studied the wrinkling deformation mechanism that occurred between two adjacent stages in multi-stage forming, which helps to optimize the deformation stages to eliminate the unnecessary wrinkling phenomenon. Through thickness analysis, numerical simulation, and fracture analysis, Chang and Chen [16] discussed the deformation mechanism of multi-stage forming parts, the way to improve the forming limit, and they also studied the elimination methods of two kinds of defects. Ndip-Agbor et al. [17] proposed a method to predict the rigid body motion in order to solve the problem of the contact between the toolpath and the sheet metal, which improved the formability of multi-stage forming. Gianluca et al. [18] formed the straight wall part by using three forming strategies: one-way incremental drawing angle, increasing the one-way incremental drawing angle of the side of the part, and the multi-directional approach with a non-horizontal path plane. The results show that the third forming strategy can redistribute the sheet metal from the thicker area to the thinner area, so that the thickness distribution is more uniform. Li et al. [19] noted that the sheet of Al 1060 had a high ductility in the normal temperature, and it was used to form aluminum parts, but could cause a large quantity of springback for formed parts. Yang et al. [20] used Al 1060 aluminum as an experimental material. The formability of sheet metal was improved by ultrasonic vibration-assisted single point incremental forming method, which effectively improved the forming limit of sheet metal. Li et al. [21] used 1060 aluminum, and Q235 and DC04 steel to make samples with different heights and analyzed the influence of materials on the dimensional accuracy of formed parts.

Ramkumar et al. [22] designed a multi-stage forming method that had better formability, a better forming angle, and better surface roughness compared with single stage forming. Zhu and Liu [23] proposed a CNC incremental forming method of multi-directional adjustment of the sheet metal posture based on virtual auxiliary body, which established a flat surface to reduce the forming angle and successfully formed the straight wall part. By changing the direction of the toolpath plane, Tanaka [24] successfully formed the straight wall parts based on the material translation controlment. In order to shorten the forming time, Wernicke et al. [25] proposed a multi-tool incremental forming method, which can improve the efficiency according to the change of toolpath and tool radius.

**Figure 1:**
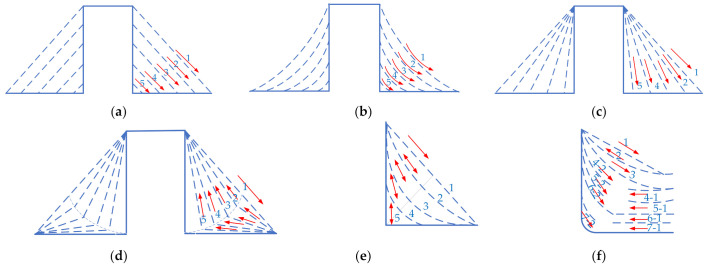
Toolpath strategies: (**a**) parallel straight line; (**b**) translation arc line; (**c**) variable angle type; (**d**) stretch-bending assisted forming; (**e**) five steps forming; and (**f**) forming based on Out-to-In (OI) toolpath.

Toolpath planning and generation is important research content of the CNC incremental forming, which has a great influence on formability and forming quality [26]. Nirala and Agrawal [27] proposed a new toolpath, which can obtain better formability, stress, and thickness distribution than the traditional toolpath, namely contour path and spiral path. Tyler and Laine [28] proposed a new toolpath, which makes the toolpath pass through the center of the workpiece rather than move along the contour, so that the thickness contour can be more uniform. Carette et al. [29] studied the toolpath and introduced a method of automatic toolpath generation based on special geometry, which can improve the surface quality of complex cross-section parts. Li et al. [30] compared and analyzed the influence of four forming toolpaths on the thickness distribution: parallel linear (Figure 1a), translational arc (Figure 1b), variable angle (Figure 1c), and stretch-bend assisted forming (Figure 1d). It was concluded that the thickness of parts formed by the variable angle forming toolpath was slightly higher than that of other toolpath strategies. It is indicated that the thinning occurs mainly at the corner in tensile-bending assisted forming. Li et al. [31] compared and analyzed the influence of three kinds of toolpath strategies of multi-stage forming, i.e., parallel linear, variable angle, stretch-bending assisted forming, and single stage forming. The authors concluded that the three toolpath strategies can effectively improve the wall thickness, in which the effect of the variable angle is slightly better. The authors further highlighted that the maximum geometric error of parallel linear is located in the transition region of the side wall and bottom surface of the part. Skjoedt et al. [32] adopted a five stage forming strategy and formed a cylindrical cup with height/radius ratio of 1 by using Down-Up-Down-Down (DUDD) and Down-Down-Down-Up (DDDU) toolpath (Figure 1e). They concluded that the thickness distribution of formed sheet parts is better than that with DDDU forming strategy. Malhotra et al. [33] proposed a new toolpath generation strategy combining the inside out and outside in forming of each intermediate stage to obtain smoother parts (Figure 1f). Bouzid et al. [34] successfully formed a forming angle of 85° through multiple stages.

In a word, researchers have done in-depth research on the forming toolpath of the straight wall parts, but most of the research objects are a straight tube and pyramid with a circular cross-section. The complex cross-section model has not been studied. Through the analysis of the above toolpath strategies, it can be seen that the thickness distribution of the straight wall parts in the parallel linear forming is more uniform, but the thickness of the surface at the top of the sheet part is relatively thin. The thickness distribution of the variable angle forming is also better, but the thickness distribution of the bottom surface is poor. The stretch-bend assisted forming is easy to deform at the corner. The combined forming strategy can effectively shorten the forming time and ensure the side wall, but it is also easy to deform at the corner and cannot guarantee the forming thickness at the bottom.

In relation to the identified problems, a new forming toolpath planning and generation method for the multi-stage incremental forming was proposed. This is based on the stretching angle, which could meet the needs for the thickness homogenization of the side wall and bottom by adjusting the position of the auxiliary line and stretching angle. It can effectively improve the thickness of the transition area between the side wall and bottom.

In the research, C++, VC++, and OpenGL library were used to develop the toolpath generation software system. Through the numerical simulation and forming experiments using 1060 aluminum sheets, the feasibility of the proposed method was verified by comparing the single stage forming, traditional multi-stage forming, and multi-stage forming based on the proposed forming toolpath.

## 2. Proposed Toolpath Planning Strategy for the Multi-Stage Forming

**Figure 2:**
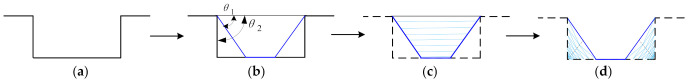
Forming strategies: (**a**) the straight wall part, (**b**) two stages forming strategy, (**c**) the first stage forming, and (**d**) the second-stage forming.

The proposed toolpath planning method for the multi-stage forming is illustrated by using the straight wall part model shown in Figure 2a as an example. For the straight wall part model, this paper adopts the two stages forming strategy as shown in Figure 2b. The purpose of the first stage forming is to reduce the forming angle and ensure the smoothness of the forming surface as much as possible, which paves the way for the second-stage forming. Therefore, the first stage forming toolpath (Figure 2c) adopts the contour toolpath.

**Figure 3:**
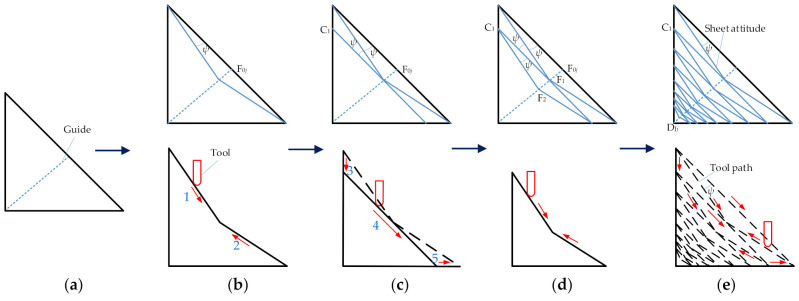
The second stage forming toolpath and forming tool movement planning: (**a**) the position of the auxiliary line; (**b**) the first layer; (**c**) the second layer; (**d**) the third layer; and (**e**) the second stage forming.

The purpose of the second stage forming is to form the side and bottom surface of the straight wall part. When planning the second stage forming toolpath, the wall thickness of the side and bottom surfaces should be as uniform as possible. Firstly, the position of the auxiliary line can be planned according to the user’s requirements for the wall thickness distribution on both sides (Figure 3a). Then, finding a suitable stretching angle *ψ*, squeeze the forming tool head from both sides to the middle (Figure 3b). Finally, extrude from top to bottom, so that the sheet metal surface after the second extrusion is parallel to the initial surface (Figure 3c). Repeat the aforementioned operations in turn (Figure 3d) to gradually form the straight wall part (Figure 3e).

The advantage of the proposed method is that the forming angle can be reduced more than with other methods. By using an appropriate stretching angle *ψ* to make the sheet surface inclined, the forming angle can be reduced more than other methods. This can effectively reduce the influence of the forming tool on the thickness of the top surface of the sheet part when the sheet is pressed down for the first time, and it helps to increase the wall thickness. At the same time, the position of the auxiliary line can be adjusted to meet the requirements of the thickness distribution of the two side walls. Because the toolpaths of each two layers are parallel and the distance between them decreases gradually, the thickness of the wall and the bottom corner can be effectively increased. In addition, the extrusion sequence of forming tool head from both sides to the middle and then from the middle to both sides can increase the thickness at the bottom corner and improve the uniformity of thickness distribution on both sides.

## 3. Forming Toolpath Generation Algorithm for Each Stage Forming

### 3.1. First Stage Forming Toolpath Generation Algorithm

Firstly, extract the inner surface of the STL model of the straight wall part and set the first stage forming angle. Then extract the upper and lower side rings of the inner surface. Offset the lower edge rings by a certain distance according to the first stage forming angle to construct the first stage forming model. Secondly, offset the first stage forming model equidistantly from the radius of the forming tool head along its normal vector direction to generate the tool location surface of the first stage forming model. Finally, equidistantly cut the tool location surface using the horizontal plane with a spacing of Δ*h* to generate the contour toolpath for the first stage forming.

### 3.2. Second Stage Forming Toolpath Generation Algorithm

**Figure 4:**
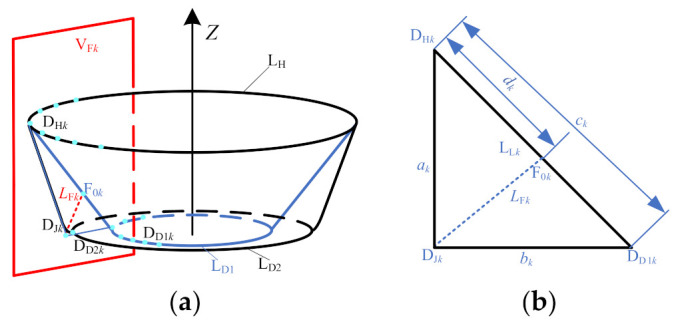
Determination of auxiliary lines: (**a**) stereogram and (**b**) longitudinal section.

**Figure 5:**
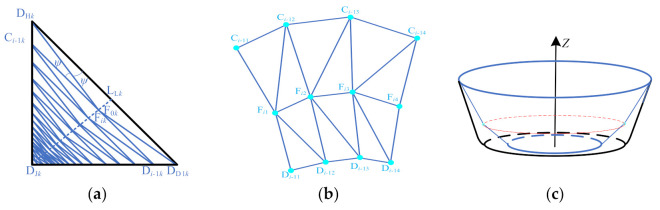
The sheet surface of the second stage forming: (**a**) longitudinal section; (**b**) triangular mesh; and (**c**) three-dimensional model of the sheet surface.

**Figure 6:**
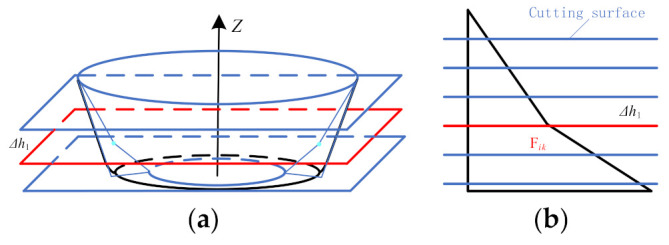
Toolpath generation method: (**a**) stereogram and (**b**) longitudinal section.

The second stage forming toolpath generation algorithm is as follows:

(1) Generate the tool location surface of the second forming stage. Offset the inner surface of the straight wall part equidistantly from the radius of the forming tool head along the direction of its normal vector to generate the tool location surface used to generate the second stage forming toolpath.
(2) Determine the position of auxiliary line L_F*k*_ (*k* represents each position on the cross-sectional contour line). Use a vertical plane V_F*k*_ passing the *Z* axis to intersect with the upper ring L_H_, lower ring L_D1_, and lower ring L_D2_ to obtain three intersection points D_H*k*_, D_D1*k*_, and D_D2*k*_, and intersect with the second stage forming model to obtain a straight wall part generatrix. Make the tangent line D_H*k*_D_J*k*_ of the generatrix to intersect the connecting lines D_D1*k*_ and D_D2*k*_ at a point D_J*k*_ and an auxiliary line L_F*k*_ in the plane V_F*k*_, as shown in Figure 4a. The distance between F_0*k*_ and point D_H*k*_, and the position of the starting point F_0*k*_ of the auxiliary line (Figure 4b) are determined by using the length ratio of the sides, namely
  $$\frac{a_{k}}{b_{k}}=\frac{d_{k}}{c_{k}-d_{k}}$$
  $$d_{k}=c_{k}\frac{a_{k}}{a_{k}+b_{k}}$$
  Here, *a_k_* is the distance between the point D_H*k*_ and the point D_J*k*_, *b_k_* is the distance between the point D_D1*k*_ and the intersecting point D_J*k*_ in the plane V_F*k*_, and *c_k_* is the distance between the connection L_L*k*_ of the point D_H*k*_ and the point D_D1*k*_. The auxiliary line L_F*k*_ is the connection line between the starting point F_0*k*_ and the intersection point D_J*k*_.
(3) Set the size of the stretching angle *ψ*. The size of the stretching angle *ψ* can be set by the user.
(4) Generate the sheet metal surface for the second stage forming. First, find the intersection point F*_ik_* (*i* represents the number of rotations of *ψ*) with each auxiliary line when the sheet material surface rotates *ψ*. For example, in the plane V_F*k*_, the line L_L*k*_ is rotated *ψ* in the F_0*k*_D_J*k*_ direction by using the point D_H*k*_ as the center of rotation, and it intersects with the auxiliary line L_F*k*_ at the point F*_ik_*. Then, take the point F*_ik_* as the center of rotation and then rotate *ψ* to intersect with the side wall at a point C_*i*-1*k*_ along the same direction, and extend the line segment C_*i*-1*k*_F*_ik_* to intersect the line segment D_D1*k*_D_J*k*_ at point D_*i*-1*k*_ (Figure 5a). Finally, the triangulation meshing is carried out on the sheet surface of each sheet posture adjusted. The method is to alternately connect the points C_*i*-1*k*_, F*_ik_*, and D_*i*-1*k*_ (Figure 5b) to construct a triangular surface to obtain the sheet surface (Figure 5c). Among them, the sheet surface of the first rotation *ψ* is obtained by constructing a triangular facet from alternately connected points D_H*k*_, F_1*k*_, and D_D1*k*_.
(5) Generate the second stage forming toolpath. A contour toolpath (Figure 6) is obtained by cutting the second stage forming sheet surface using a horizontal plane with a layer spacing of Δ*h*_1_ equally from the horizontal plane where the point F*_ik_* is located. In addition, finally obtain the second forming toolpath by determining the moving direction of the forming tool according to the moving sequence of the forming tool.

### 3.3. Case Studies

**Figure 7:**
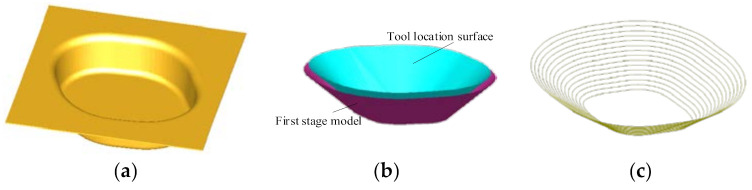
First stage forming toolpath: (**a**) the straight wall part model; (**b**) the first stage forming model and the tool location surface; and (**c**) the first stage forming toolpath.

In order to verify the feasibility of the forming toolpath planning and generation algorithm, a software system of forming toolpath planning and generation is developed by using C++ language, OpenGL graphics library, and VC++ 6.0 in the Windows 7 environment. The straight wall part model shown in Figure 7a (forming angle is 80°, forming depth is 22 mm, the arc radius at the bottom is 6 mm) is taken as an example model, and a case study of forming toolpath planning and generating algorithm is given.

Figure 7b shows the tool location surface of the first stage forming after the first stage forming model is offset by a tool head radius distance in the direction of the normal vector of the inner surface. Figure 7c shows the contour toolpath with equal distance when the layer distance Δ*h* is set as 1 mm and the first stage forming angle is set as 45°.

**Figure 8:**
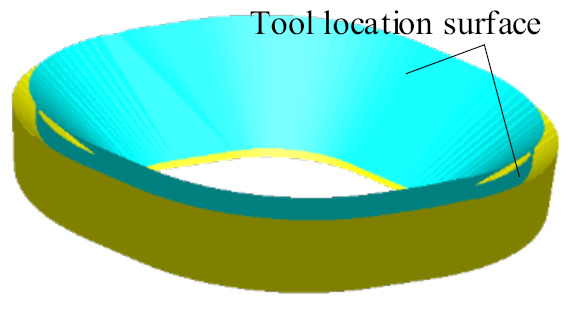
The second stage forming area.

Figure 8 shows the second stage forming area, which is composed of the tool location surface (Figure 7b) of the first stage forming model and the surface on which the inner surface of the straight wall part is offset from the radius of the tool head in the direction of its normal vector.

**Figure 9:**
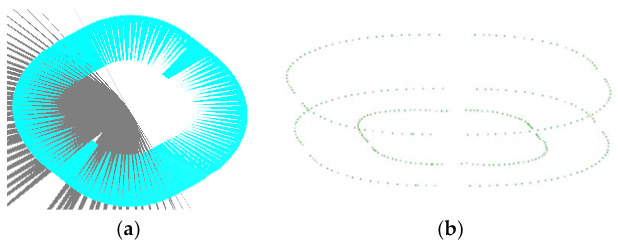
The cutting process: (**a**) vertical plane and (**b**) points.

Figure 9a shows the process of cutting the second stage forming area with vertical planes. Figure 9b shows the points on the edge ring of the second stage forming area obtained after cutting. 

**Figure 10:**
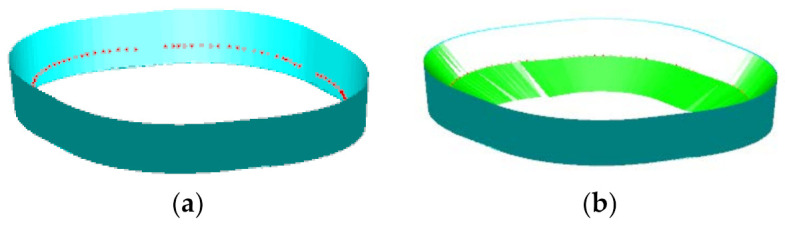
The sheet surface generation: (**a**) starting points of auxiliary line and (**b**) the inclined sheet surface of the first layer.

Figure 10a shows the starting point of the auxiliary line (that is, the position of F_0*k*_). Figure 10b shows the inclined sheet surface of the first layer when the stretching angle *ψ* is 15°. It is obtained by alternately connecting points D_H*k*_, F_1*k*_, and D_D1*k*_ to construct a triangular surface.

**Figure 11:**
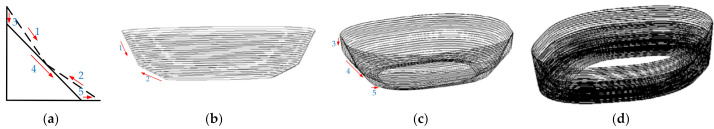
The second stage forming: (**a**) the toolpath schematic of the first two layers; (**b**) toolpath of the first layer; (**c**) toolpath of the second layer; and (**d**) the second stage forming toolpath.

Figure 11 shows the second stage forming toolpath when the stretching angle *ψ* is 15° and the layer distance Δ*h*_1_ is 1 mm. Figure 11a shows the toolpath schematic of the first two layers. Figure 11b shows the forming toolpath of the first layer, where the forming tool moves first from top to bottom and then from bottom to top. Figure 11c shows the forming toolpath of the second layer. After the first layer toolpath forming is completed, the forming tool moves from top to bottom, then from top to bottom, and then from left to right. Figure 11d shows the forming toolpath the second stage forming, which consists of many layers.

## 4. Finite Element Analysis

**Figure 12:**
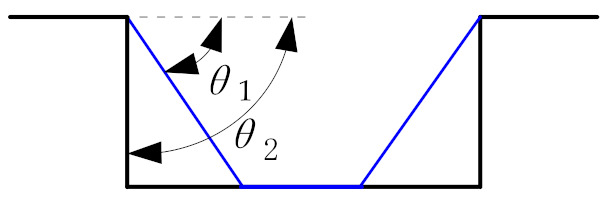
Traditional multi-stage incremental forming strategy.

In order to verify the feasibility of the proposed forming toolpath generation method, the traditional multi-stage forming (TMSTF) and the proposed multi-stage forming (PMSF) are used to simulate the forming process of the sheet metal part as shown in Figure 7a by using ANSYS/LS-DYNA software (19.0, ANSYS, Pittsburgh, PA, USA). The thickness distribution and strain of sheet are compared and analyzed. In the numerical simulation analysis, the spindle speed is set to 400 rpm and the feed rate is set to 600 mm min^−1^. The traditional multi-stage forming and the proposed multi-stage forming in this paper adopt the two stage forming strategy shown in Figure 2 and Figure 12, respectively. The first stage forming angle *θ*_1_ and the second stage forming angle *θ*_2_ are 45° and 80°, and the first stage forming toolpath adopts the traditional contour toolpath with a layer distance of 1 mm.

In the second stage forming, the traditional multi-stage incremental forming still adopts the traditional contour toolpath with a layer distance of 1 mm. The proposed multi-stage forming in this paper adopts the forming toolpath shown in Figure 3, with the layer distance of 1 mm. The stretching angle *ψ* is set to 5° and 15°.

### 4.1. Establishment of the Finite Element Analysis Model

In the numerical simulation, the analysis process adopts the Belytschko–Wong–Chiang algorithm. The sheet is a 1060 aluminum sheet with the size of 200 mm × 200 mm × 1 mm and its element type is a SHELL 163 shell unit. The forming tool is set as a ball head tool with a diameter of *Φ*10 mm that is made of W6Mo5Cr4V2 high speed steel, and its element type is a SOLID 164 body unit. The support adopts GCr15 bearing steel and its element type is SOLID 164 body unit. Both the support and the forming tool are set as rigid body. The mechanical property parameters of each material are shown in Table 1.

The support, the sheet, and the forming tool head are meshed, respectively. The support is meshed by 4 mm free grid method, the number of elements of the mesh is 11,641. The sheet is meshed by 1.5 mm mapping method, the number of elements of the mesh is 17,956. Finally, the forming tool head is meshed by 1.5 mm free grid method, the number of elements of the mesh is 1260. The friction between the support, sheet, and tool head is defined. The contact between the tool head and sheet is nodes to surface contact, in which the tool head is the contact surface and the sheet is the target surface. The static friction coefficient and dynamic friction coefficient are set as 0.1 and 0.05, respectively. The contact between the support and the sheet is surface to surface contact, in which the support is the target surface and the sheet is the contact surface. The static friction coefficient and dynamic friction coefficient are set as 0.5 and 0, respectively. The constraints of the model are defined by restricting the movement of the four sides of the sheet and the *X*, *Y*, and *Z* axes of the supporting die, the six degrees of freedom of rotation around the *X*, *Y*, and *Z* axes, and restricting the degrees of freedom of the extrusion tool head to rotate around the *X*, *Y*, and *Z* axes.

### 4.2. Finite Element Analysis Results

**Figure 13:**
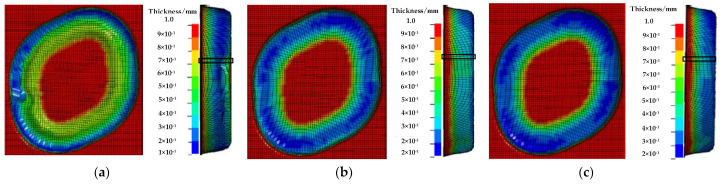
The thickness distribution: (**a**) the traditional multi-stage; (**b**) the proposed method (*ψ* = 5°); and (**c**) the proposed method (*ψ* = 15°).

The simulation results between the traditional multi-stage incremental forming and the proposed multi-stage incremental forming are compared by three aspects: thickness distribution, strain curve, and sheet metal flow. Figure 13a shows the thickness distribution nephogram obtained by numerical simulation of straight wall part using the traditional multi-stage forming strategy, and its thickness distribution range is (0.1788, 0.9996). Figure 13b,c shows the thickness distribution nephogram obtained by numerical simulation of the proposed multi-stage forming with *ψ* = 5° and *ψ* = 15° stretching angles, and their thickness distribution ranges are (0.2178, 0.9869) and (0.2765, 0.9852). By comparing and analyzing the thickness distribution nephograms it can be concluded that the thickness distribution of the proposed multi-stage incremental forming is better than that of the traditional multi-stage incremental forming. The bottom thickness distribution is more uniform, and the bulge phenomenon is obviously reduced; the stretching angle also has some influences on the thickness distribution. The stretching angle of 15° is slightly better than that of 5°, and the minimum thickness difference of them is 0.0587. As can be seen in Figure 13, the thickness distribution of the proposed method is more uniform than that of the traditional multi-stage incremental forming.

**Figure 14:**
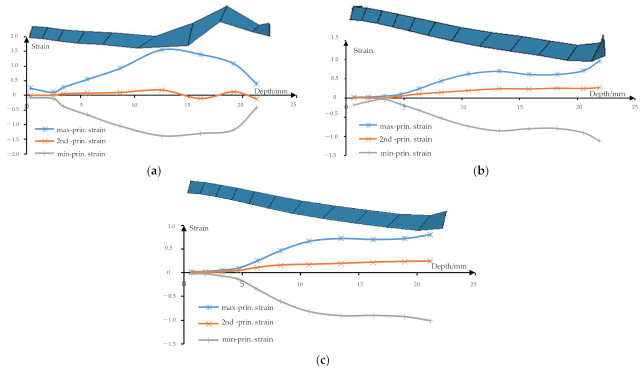
The strain curves: (**a**) the traditional multi-stage; (**b**) the proposed method (*ψ* = 5°); and (**c**) the proposed method (*ψ* = 15°).

Figure 14 shows the strain curves that are drawn according to the strain data of the elements extracted in the side wall area shown in Figure 13. First, extract the cells in the sidewall shown in Figure 13 and then extract the height and strain values of each corresponding cell in the area. Finally, draw the strain curves using the Excel software. It can be seen from the strain curve of traditional multi-stage incremental forming shown in Figure 14a, with the increase in depth, the first principal strain increases sharply, the second principal strain basically remains close to 0, and the third principal strain also changes continuously. That is, with the increase in depth, the vertical dimension of cells increases further, the transverse dimension of cells is almost unchanged, and the thickness of cells decreases further, which leads to the decrease in the thickness of the sheet with the increasing of depth. The horizontal flow direction of cells near the depth of 18 mm is also changed. It can be seen from the multi-stage incremental forming shown in Figure 14b (*ψ* = 5°), with the increase in depth, the first principal strain increases slowly, the second principal strain also increases slowly, which makes the third principal strain increase slowly. That is, with the increase in depth, the vertical dimension of cells increases slowly, and the horizontal dimension increases slowly, and the thickness of cells decreases slowly. Figure 14c shows the strain curve when the stretching angle *ψ* is 15°. With the increase in depth, the first principal strain increases slowly, and the second principal strain also increases slowly, which makes the third principal strain also increase slowly. When the depth is close to 22 mm, the first principal strain and the second principal strain with the stretching angle *ψ* is 5° are larger than that with the stretching angle *ψ* is 15°, which makes the third principal strain decrease more greatly, and thus the thickness decreases more greatly.

**Figure 15:**
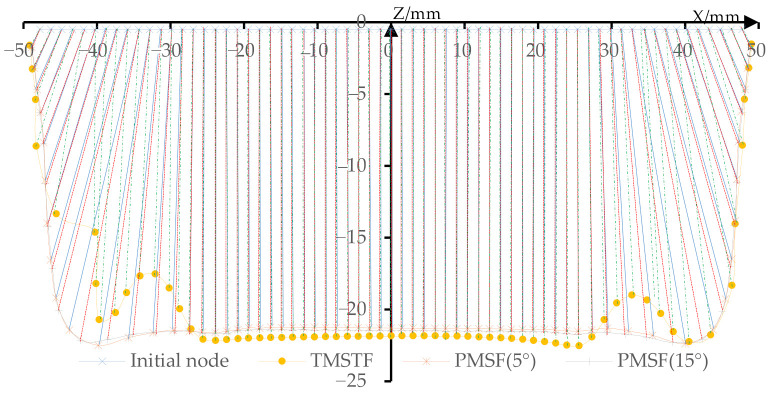
The material flow.

In order to study the material flow of the multi-stage forming and the proposed multi-stage forming (*ψ* is 5° and 15°), the material flow curves shown in Figure 15 are drawn by using the coordinates of the nodes on the *Y* = 0 section of the design model and the formed parts. The material flow range of traditional multi-stage forming is relatively large, there is bulge phenomenon in the range of (−39, −27) and (27, 39), and there is subsidence phenomenon in the range of (−28.5, 28.5). Using the sheet metal flow in (42, 48) region as an example, it can be seen from the relative position of two adjacent nodes that the distance between the two nodes of the formed part gradually increases with the increase in depth, that is, the thickness of the sheet in (42, 48) region gradually decreases with the increase in depth. The proposed multi-stage forming with stretching angle 5° has no bulge phenomenon, but there is subsidence phenomenon in the range of (−28.5, 28.5), which is smaller than the whole subsidence in the multi-stage forming. Within the interval of (42, 48), the distance between two adjacent nodes gradually increases with the increase in depth, and the growth rate is relatively slow, that is, the thickness decreases relatively slowly. In the traditional multi-stage forming, less material flows to the side wall, which results in the sinking and bulging. However, the material in the proposed multi-stage forming is more evenly distributed in the side wall and bottom.

**Figure 16:**
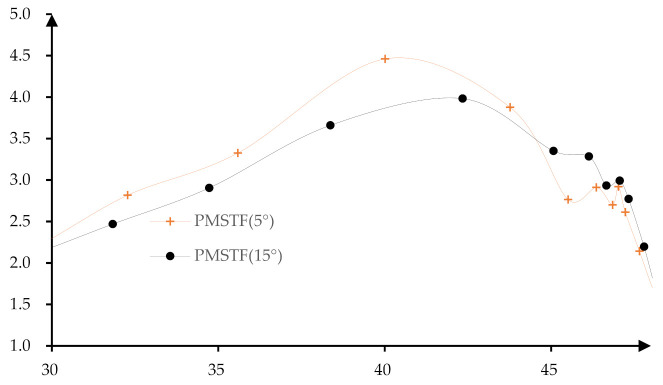
Distance between two adjacent nodes.

By comparing the material flows of the stretching angle of 5° and 15°, the sheets participating in the material flow are the same and the contours of the formed part are similar. For the convenience of analysis, the nodes at (30, 48) are extracted and the distance between two adjacent points is calculated and drawn into a curve (Figure 16). However, it can be clearly found that the distance between the two nodes with a stretching angle of 5° is smaller first and then changed to be larger than that with the stretching angle of 15°. This causes the thickness to be changed from large to small, and the thickness of the transition area from the side wall to the bottom is small, so that the thickness distribution of the stretching angle of 15° is relatively uniform.

## 5. Forming Experiment

### 5.1. Experimental Process

In this paper, the single stage forming, traditional multi-stage forming, and multi-stage forming based on the proposed forming toolpath generation method are respectively used to form the straight wall part shown in Figure 7a. The feasibility of the proposed forming toolpath generation method is evaluated by comparing and analyzing the thickness distribution, forming depth, and the contour curve of the formed sheet metal parts obtained by three forming strategies.

In the forming experiment, the machine tool uses the Roland 3D mold engraving machine MDX-540 (Roland DG Corporation, Osaka, Japan) and the sheet uses 1060 aluminum sheet with a size of 200 × 200 × 1 mm^3^. The forming tool is a hemispherical forming tool with a diameter of 10 mm and the material of the support is a chemical wood. The single stage forming, the traditional multi-stage forming, and the proposed multi-stage forming all adopt contour toolpath with a layer distance of 0.2 mm. The spindle speed is set to 400 rpm and the feed rate is set to 600 mm min^−1^. 

**Figure 17:**
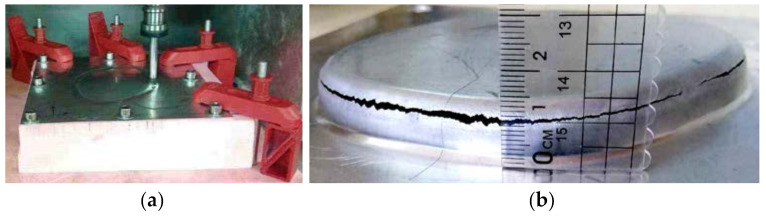
The single stage forming: (**a**) the forming process and (**b**) the formed part.

Figure 17a shows the process of the forming experiment with single stage forming method strategy. When the forming experiment reached a depth of 9 mm, the sheet metal had been broken (Figure 17b), which indicates that the straight wall part cannot be formed by single stage forming.

**Figure 18:**
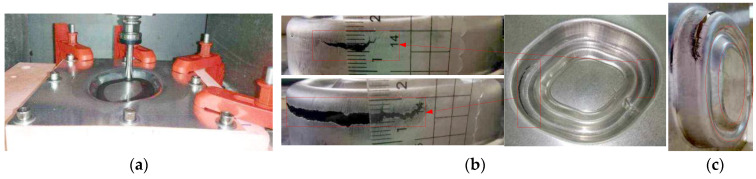
The traditional multi-stage forming: (**a**) the forming process; (**b**) the formed part; and (**c**) the bottom.

Figure 18a shows the forming process with the traditional multi-stage forming strategy (shown in Figure 12). Figure 18b shows the sheet part obtained by the traditional multi-stage incremental forming strategy. It can be seen that the appearance quality of traditional multi-stage incremental forming is poor: two cracks appear in the side wall at the depth of 16 mm and 17 mm, respectively, and serious bulging phenomenon appears at the bottom (Figure 18c).

**Figure 19:**
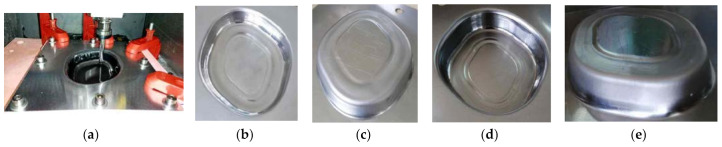
The proposed multi-stage forming: (**a**) the forming process, (**b**) the forward view of the straight wall part (*ψ* = 5°), (**c**) the reverse view of the straight wall part (*ψ* = 5°), (**d**) the forward view of the straight wall part (*ψ* = 15°), and (**e**) the reverse view of the straight wall part (*ψ* = 15°).

Figure 19 shows the forming process and the formed parts obtained by using the multi-stage forming based on the proposed forming toolpath (Figure 3). Among them, Figure 19a shows the forming process, and Figure 19b,c shows the forward and reverse views of the straight wall part formed with a stretching angle *ψ* = 5°. Figure 19d,e shows the forward and reverse views of the straight wall part formed with a stretching angle *ψ* = 15°. It can be seen that the appearance quality of these two formed parts is good, the side walls are smooth, and the bottom bulging phenomenon is obviously eliminated compared with the traditional multi-stage forming strategy.

### 5.2. Forming Experimental Results

Since the formed parts using single stage forming and traditional multi-stage forming strategies are cracked, the two formed parts are not measured and analyzed. However, only two formed parts with proposed multi-stage forming (*ψ* = 5° and *ψ* = 15°) are compared and analyzed for the dimensional accuracy of profile, thickness distribution, and surface roughness.

#### 5.2.1. Dimensional Accuracy of Profile

**Figure 20:**
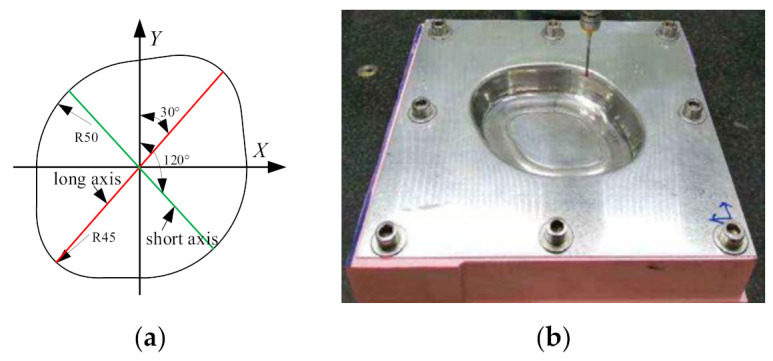
Profile measurement: (**a**) measurement position and (**b**) measurement process.

In order to compare the difference between the profile curves of the formed part and the theoretical model, the Hexagon three coordinate measuring instrument is used to measure the profile along the sections of the long axis and the short axis of the sheet (Figure 20a) with the measurement distance of 0.1–1 mm. Figure 20b shows the process of measuring the sheet part with a three coordinate measuring instrument.

**Figure 21:**
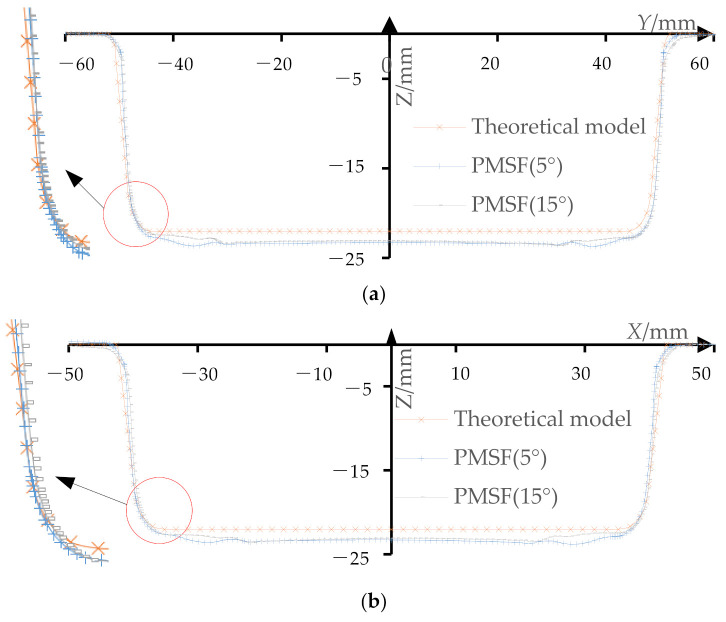
The profile curve: (**a**) the section of the long axis and (**b**) the section of the short axis.

Import the measured data into Excel software and draw the profile curve of the long axis section (Figure 21a) and the short axis section (Figure 21b). It can be seen that the transition region from side wall to the bottom of the formed part of the proposed multi-stage incremental forming is fit well with that of the theoretical model, but it has a slight sinking phenomenon compared with the theoretical model. When the stretching angle *ψ* = 5°, the subsidence of profile section along where the long axis is located mainly lies in the range of *Y* coordinate (−38, 38), and the subsidence of profile section along where the short axis is located mainly lies in the range of *X* coordinate (−31, 31). When the stretching angle *ψ* = 15°, the subsidence of profile section along where the long axis is located mainly lies in the range of *Y* coordinate (−31, 33), and the subsidence of profile section along where the short axis is located mainly lies in the range of *X* coordinate (−22, 22).

**Figure 22:**
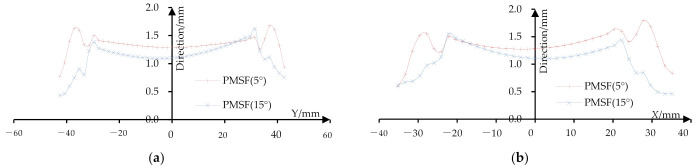
*Z*-direction deviation of the profile curves: (**a**) the section of the long axis and (**b**) the section of the short axis.

In order to compare and analyze the influence of two different stretching angles (*ψ* = 5° and *ψ* = 15°) on the dimensional accuracy of the profile of the formed part, the *Z* direction deviation of the profile of the section with the long axis (Figure 22a) and the *Z* direction deviation of the section with the short axis (Figure 22b) are drawn.

The maximum and mean deviations of the profile curves in *Z* direction are shown in Table 2. When the stretching angle is 5°, the maximum *Z* direction deviation of the profile curves at the section on the long axis is at *Y* = −37 position and its value is 1.6759. The *Z* direction deviation is mainly distributed in the range of (1.25, 1.45) and its average value is 1.3160. The maximum *Z* direction deviation of the profile at the section on the short axis is at *X* = 28 position and its value is 1.7947. The *Z* direction deviation is mainly distributed in the range of (1.25, 1.45) and its average value is 1.3455. When the stretching angle is 15°, the maximum *Z* direction deviation of the profile at the section on the long axis is at *Y* = 31.5 position and its value is 1.6212. The *Z* direction deviation is mainly within the range of (1.05, 1.20) with average value of 1.1219. The maximum *Z* direction deviation of the profile at the section on the short axis is 1.5517 at *X* = −22 position and the *Z* direction deviation is mainly distributed in the range of (1.10, 1.25) with an average value of 1.0706. From the above analysis, it can be seen that when the stretching angle is 15°, the dimensional accuracy of the profile of the formed part is higher than that when the stretching angle is 5°.

#### 5.2.2. Thickness Distribution

**Figure 23:**
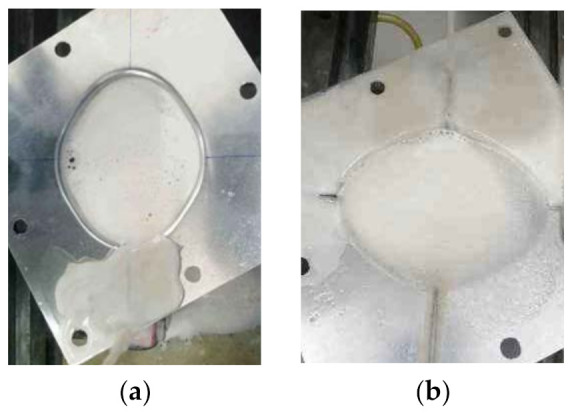
Cutting process: (**a**) the long axis direction cutting and (**b**) the short axis direction cutting.

In order to analyze the thickness distribution of the formed part on the two longitudinal sections along the direction of the long axis and the short axis shown in Figure 20a, the formed part is cut along the long axis and the short axis by wire cutting machine. Figure 23 shows the process of wire cutting. 

**Figure 24:**
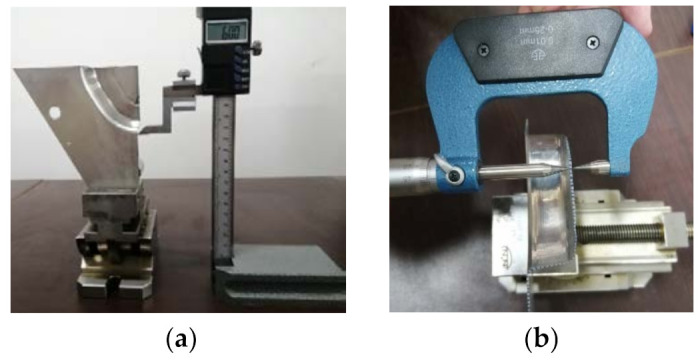
Thickness measurement: (**a**) sample point marking and (**b**) thickness measurement.

**Figure 25:**
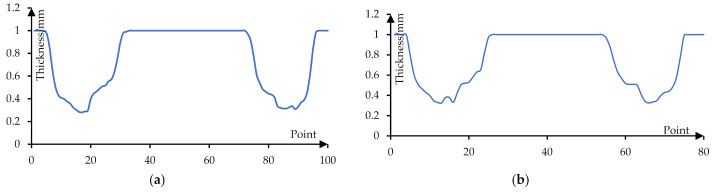
The thickness distribution curve (5°): (**a**) the thickness of the long axis section and (**b**) the thickness of the short axis section.

**Figure 26:**
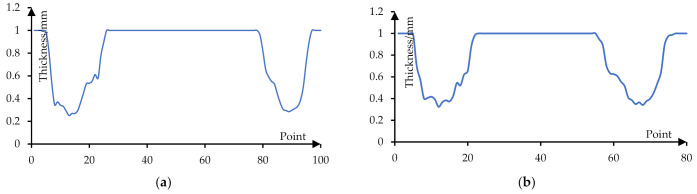
The thickness distribution curve (15°): (**a**) the thickness of the long axis section and (**b**) the thickness of the short axis section.

In order to measure the thickness of the sheet metal, the height gauge is used to mark the sample points with a distance of 2 mm on the cutting surface of the formed part (Figure 24a). The thickness at the sample points is measured by using a pointed micrometer (Figure 24b) and the measurement accuracy is 0.001 mm. The data is imported into Excel software and the thickness distribution curves are drawn (Figure 25 and Figure 26). It can be seen that as the depth increases, the thickness gradually decreases.

Figure 25 shows the thickness distribution curve of formed parts when the stretching angle is 5°. Among them, Figure 25a shows the thickness distribution of the formed part on the long axis section. It can be seen that the thickness of the transition area from the side wall to the bottom of the formed part is relatively thin and its minimum value is 0.28 mm. Figure 25b shows the thickness distribution of the formed part on the short axis section and its minimum value is 0.325 mm.

Figure 26 shows the thickness distribution curve of the formed part when the stretching angle is 15°. Figure 26a shows the thickness distribution of the formed part on the long axis section and its minimum value is 0.29 mm. Figure 26b shows the thickness distribution of the formed part on the short axis section and its minimum value is 0.324 mm. 

Table 3 shows the minimum thickness distribution. It can be seen from Table 3 and Figure 25 and Figure 26 that the sheet thickness of the long axis section is slightly smaller than that of the short axis section. The sheet thickness of the formed part with a stretching angle of 5° decreases more slowly than that of the formed part with a stretching angle of 15°, but the thickness of the formed part with a stretching angle of 15° is thicker than that with a stretching angle of 5°.

#### 5.2.3. Roughness

**Figure 27:**
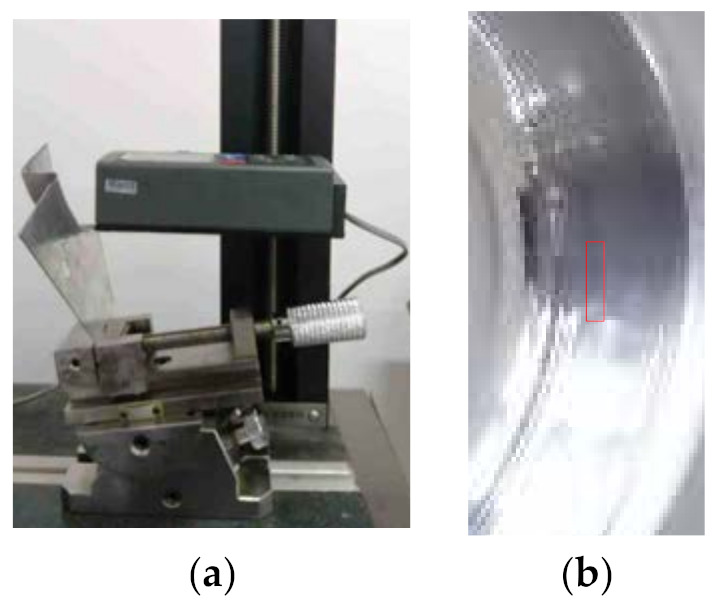
The roughness measurement: (**a**) measurement process and (**b**) measurement area.

The roughness of the formed parts is measured by a TIME 3200 roughness instrument. Figure 27a shows the roughness measurement process in which the roughness of the box area shown in Figure 27b is measured.

**Figure 28:**
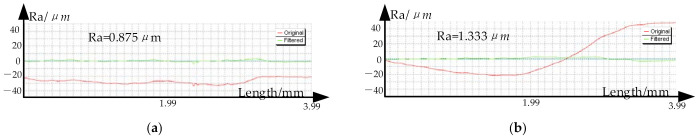
The roughness curves: (**a**) *ψ* = 5° and (**b**) *ψ* = 15°.

Figure 28a,b shows the roughness curves of formed parts based on when the stretching angle is 5° and the stretching angle is 15°, respectively. It can be seen that the surface roughness of the stretching angle 5° is better that of the stretching angle 5° in this area.

### 5.3. Discussion and Analysis

In summary of the data analysis described above, because the second stage forming in traditional multi-stage forming has a larger forming angle and a larger material flow on the sidewalls, this causes the thickness to be thinned and leads to the sheet rupture. 

In addition, due to too much material flow to the bottom, this results in the bottom bulging and sinking. In the proposed method, the forming angle of the second stage forming is reduced by building a flat surface so that the sheet material flows evenly to the bottom and sidewall. When the *X* coordinate is within the range of (46, 48), the sheet materials flow of the stretching angle 5° is about 0.247 mm smaller than that of the stretching angle 15°. However, when the *X* coordinate is within the range of (30, 44), the sheet materials flow of the stretching angle 5° is about 0.269 mm larger than that of the stretching angle 15°. That is, the thickness of the formed part decreases more slowly in the range of (46, 48), and the thickness of the formed part decreases faster in the range of (30, 44), which results in a less uneven thickness distribution.

## 6. Conclusions

(1) The straight wall parts with the forming angle of 80° cannot be formed by the single stage forming, in which the sheet metal has been cracked when the forming depth reaches 14 mm.
(2) The traditional two stage incremental forming cannot form the straight wall parts with the forming angle of 80°. There are two cracks on the side wall at the depth of 16 mm and 17 mm, and serious bulging occurs at the bottom.
(3) The straight wall parts with the forming angle of 80° can be formed by using the proposed multi-stage incremental forming strategy. Although there is a slight subsidence at the bottom, the profile of the side wall becomes closer to that of the target model with the increase in depth. By analyzing the thickness distribution of the section in the direction of the long axis and the short axis of the straight wall parts, it can be concluded that the thickness decreases gradually with the increase in depth. The thickness of the long axis is smaller than that of the short axis, and the minimum thickness is 0.28 mm.
(4) Different stretching angles have different effects on the thickness and contour of the formed parts. In this paper, the stretching angle of 5° and 15° were respectively used to carry out the forming experiments. It is concluded that the profile size accuracy of the stretching angle 15° is higher and the bottom subsidence of the stretching angle 15° is smaller, while the thickness of 5° is reduced slowly, but the thickness of the stretching angle 15° is thicker. In the area near the bottom, the surface roughness at a stretching angle of 5° is better.
(5) In future research, it will be necessary to further study the influence of the stretching angle, moving direction on the forming quality, and process parameter optimization methods. In addition, the influence of the different auxiliary line positions on the thickness distribution and the influence of force, material properties, and springback on the forming quality are needed to be studied.
(6) The research in this paper provides a new forming toolpath for the multi-stage incremental forming, which can successfully form the straight wall parts and prompt the development of the incremental forming of straight wall parts.

## Figures and Tables

**Table 1:** Material mechanical property parameters.

Material	Density [kg·m^−3^]	Elastic Modulus [GPa]	Poisson’s Ratio	Yield Stress [MPa]	Tangent Modulus [GPa]	Hardening Coefficient
Al1060	2700	55.94	0.324	153.6	2.9	0.198
GCr15	8160	218	0.30	-	-	-
W6Mo5Cr4V2	7810	212	0.29	-	-	-

**Table 2:** Maximum and mean deviation.

Stretch Angle	Maximum Deviation at Long Axis [mm]	Mean Deviation at Long Axis [mm]	Maximum Deviation at Short Axis [mm]	Mean Deviation at Short Axis [mm]
5°	1.6759	1.3160	1.7947	1.3455
15°	1.6212	1.1219	1.5517	1.0706

**Table 3:** Minimum thickness distribution.

Stretch Angle	Minimum Thickness at Long Axis [mm]	Minimum Thickness at Short Axis [mm]
5°	0.280	0.325
15°	0.290	0.324

## Data Availability

Not applicable.
